# The effects of social connections on self-rated physical and mental health among internal migrant and local adolescents in Shanghai, China

**DOI:** 10.1186/1471-2458-12-97

**Published:** 2012-02-03

**Authors:** Zheng-hong Mao, Xu-dong Zhao

**Affiliations:** 1 Department of Psychiatry, Tongji University School of Medicine, 1239 Siping Road, Shanghai 200092, China; 2 Institute of Psychosomatic Medicine, Shanghai East Hospital, Tongji University, 150 Jimo Road, Shanghai 200120, China

## Abstract

**Background:**

China is in the midst of history's largest flow of rural-urban migration in the world; a flow that includes growing numbers of children and adolescents. Their health status is an important public health issue. This study compares self-rated physical and mental health of migrant and local adolescents in China, and examines to what extent layered social connections account for health outcomes.

**Methods:**

In 2010, we conducted a cross-sectional study among middle school students in Pudong New Area, Shanghai. Information about health status, social connections, and demographic factors were collected using a questionnaire survey. After controlling for sociodemographic factors, we used the *t-*test, Chi-square analysis, and a series of regression models to compare differences in health outcomes and explore the effects of social connections.

**Results:**

Migrant adolescents reported significantly higher rates of good physical health. However, they also had significantly fewer social connections, lower self-esteem, and higher levels of depression than their native peers. Family cohesion was associated with depressive symptoms and low self-esteem among all adolescents; peer association and social cohesion played major roles in migrants' well-being. Gender, age, and socioeconomic (SES) factors also affected adolescents' self-rated physical and mental health.

**Conclusions:**

Self-rated data suggest that migrant adolescents enjoy a physical health advantage and a mental health disadvantage. Layered social connections, such as peer association and social cohesion, may be particularly important for migrants. A public health effort is required to improve the health status of migrant youth.

## Background

According to the 2009 census in China, there were 211 million internal migrants and among them, more than 78% were rural-to-urban migrants [1]. Since the early 1990s, one of vital changes of migration pattern is from individual to family migration [1,2]. For example, from 1993-1997, the size of the floating population remained stable in Shanghai, but the number of migrant children of school age increased from 280,000-340, 000 [3]. At present, approximately 20.8% of Chinese internal migrants are school-aged children (not above 14 years old), equivalent to approximately 43.89 million individuals who have accompanied their parents in relocating to cities [1].

Migration and migration-related processes have been widely assumed to affect the health of children and adolescents [4], especially rural-to-urban migration [5,6]. For example, vaccination and regular health examination rates among migrant children and adolescents are reported to be significantly lower than the average for their urban peers [7]. In addition to lack of health insurance, access to care may be limited by parental lack of knowledge of health care needs and resources [7,8]. Studies that focus on the mental health status of Chinese migrant children and adolescents generally show disadvantages, such as lower self-esteem [9]; higher loneliness, social anxiety, and perceived discrimination [10]; and more behavioral problems [11].

Prior reports indicate that low socioeconomic status (SES), social exclusion, and fragmentation of social support networks may lead to poor self-esteem, depression, and anxiety in immigrant populations [12-14]. The relationship between social connections and individual well-being has long been recognized in Western literature. It dates back to the work of Durkheim [15], who documented a link between social isolation and reduced psychological well-being. Since then, a wide range of studies have identified beneficial effects from social networks (including family, friends, neighbors), and social cohesion on individual health [16-18]. Zhang and colleagues [18] identified three layers of social connections-the inner layer of family cohesion, the intermediate layer of support by friends and relatives, and the outer layer of social cohesion. Several surveys report that these layers are differentially related to better health, and may have significant effects on migrants' health [16,18]. For instance, family cohesion has independent and direct effects on both self-rated physical and mental health among Asian Americans. However, the impact of other social connection measures is partially mediated by SES and immigration-related factors [18].

A large body of evidence suggests that important relationships for adolescents usually fall into the following domains: family, peer group, school, and the community and society at large [19,20]. The quality of family relationships is central to development during childhood and adolescence. Harker [21] found that migrant children and adolescents who felt closer to their parents tended to score higher on various measures of psychological adjustment, and suffer few adverse effects from stressful events. Data indicate that relationships with peers are also one of the most important factors in the social adaptation of migrant adolescents [22]. In the school domain, a teacher-student relationship is very influential in the development of school-aged children and adolescents. In addition, schools can strengthen social and cultural capital, especially among immigrant pupils [23]. In the social cohesion domain, a social network and perceived social support are also related to adolescents' inner and outer problems, loneliness, school adaptation [24], and self-esteem [9,25].

Based on prior research [18-20], we differentiated adolescents' social connections into four types-family cohesion, peer association, teacher-student relations, and social cohesion. However, there are few studies on the relation of Chinese migrant adolescents' multiple social connections and health. Most often, reports focus largely on migrants' limited resources, e.g., poor living conditions, limited access to urban health care, and vulnerability to infectious diseases, including HIV and STDs [26,27]. To date, scant data exist on the health effects of migration on Chinese adolescents. In addition, little empirical work has simultaneously examined multiple social connections in relation to the health of migrant adolescents.

The aims of this study are to compare the self-rated physical and mental health of migrant adolescents and native peers; examine the effects of different types of social connections on adolescents' self-rated health; explore whether the effects of layered social connections vary according to an individual's migrant status; and examine whether SES and demographic factors (e.g., gender, family income, parental education) affect the health of migrants differently than that of local adolescents.

## Methods

### Design, sample, and setting

This study was carried out in Pudong New Area of Shanghai, which has a high proportion of migrants in the population. In 2004, Shanghai was the largest host city for internal migrants in mainland China, and accommodated 3.87 million migrant workers from other parts of the country [28]. Residents in Pudong New Area represent diverse occupations and backgrounds. Also, suburban schools in Shanghai, typically serve children of the general population in contrast to some of the "key schools" (located in the city proper) that cater only to elite and gifted children [29]. These factors make suburban public schools well-suited to the study of Chinese migrant adolescents, especially those who have moved from rural to urban areas.

The sampling process was as follows: 1) three regular middle schools were randomly selected from the suburban districts of Pudong New Area; and 2) four classes in each grade, from grades 6-8, were randomly chosen from the schools. Anonymous, self-administered questionnaires were administered from September to November 2010. All students received the same instructions; questionnaires were collected at the end of the class session. All participants were informed of the study's purpose; written consent from students and parents were acquired. The project was reviewed and approved by the Institutional Review Board of Tongji University.

### Measures

#### Dependent variables

##### Self-rated physical and mental heath

Our analysis of mental health focused on self-esteem and depression. The general self-esteem was measured by 10-item instrument developed by Rosenberg [30] (Cronbach's alpha = 0.77). Depressive symptoms were measured with a 20-item modified version of the Center for Epidemiological Study Depression scale (CES-D) [31] (Cronbach's alpha = 0.81). Data show that the two instruments have high reliability and internal consistency in various populations of adolescents [20,32]. Self-rated physical health was measured by responses to the question, "How would you rate your overall physical health, as excellent, very good, good, fair, or poor?" The variable was generally dichotomized into excellent/very good/good versus fair/poor. These three measures have well-documented construct and criterion validity, and have been validated in multiple sites, including China [17,25,33].

#### Independent variables

##### Social connections

Social connections were measured using four indices, including family cohesion, peer association, teacher-student relations, and social cohesion. All measures were average indices with higher scores reflecting higher levels of social connection. Family cohesion was measured by 15 items selected from the Family Adaptability and Cohesion Scale (FACES-II) [34]. Cohesion is defined as the emotional bonding that family members have towards one another, and the degree to which family members are connected. The FACES-II-CV was demonstrated to have high internal consistency, with Cronbach's alpha of perceived cohesion being 0.85 [35]. Results support the discriminant validity of FACES-II for Chinese families. Peer association, and teacher-student relations were assessed by 6 and 5 items, respectively, from the School Adaption Scale [36], a 27-item scale that assesses student adaption in school life contexts. The 11 selected items were determined by factor analysis of the component items that reflect connection with peers and teachers. Respondents were asked to rate the frequency of certain peer and teacher-student interactions in schools on a five-point Likert-type scale ranging from (1) very strongly disagree to (5) very strongly agree. Representative items included such statements as "I have friends I can depend on, and I think my teachers can understand me." Cronbach's alpha coefficients were 0.82 and 0.71 for the respective subscales in this study. Social cohesion was measured by an 8-item modification of the Social Support Rate Scale (SSRS) [37]. Selected items assess adolescents' ability to rely on adults, friends, neighbors, or organizations for support. They also evaluate the degree to which adolescents feel that adults and friends care about them. Response categories ranged from (1) strongly disagree to (4) strongly agree. Representative items include: I will count on friends when things go wrong; my relatives are willing to help me make decisions; I have always actively participated in school or community activities. In this study, the Cronbach's alpha coefficient was 0.76.

##### Socioeconomic Status (SES)

Several contextual factors were assessed that are believed linked to adolescents' SES, such as monthly household income (RMB) (< 3,000; 3,000-4,999; 5,000-10,000; > 10,000), and parental education (middle school or less; high school to some college; college and greater). The status of parental occupation was categorized as low (farmers, machine operators, service workers, and laborers), middle (clerical workers, sales workers, and craftsmen), and high (professionals, physicians, and managers) [38].

##### Demographic variables

Gender, age, migration status (with or without registered Shanghai residency), and location of residence were also examined. The latter was categorized into village, suburb, town and city, and then dichotomized into village/suburb vs. town/city.

#### Data analysis

We performed the *t*-test and *Chi*-square test to compare migrants and local adolescents, and used logistic models to control for potential confounders and test associations between the binary results of self-rated excellent/good physical health and independent variables. Adjusted odds ratios (OR) and 95% confidence intervals (95% CI) were calculated. We also used a series of linear regression models to test self-esteem and depressive mood. We conducted regression analyses of each health outcome among the total sample (Model 1), migrant sample (Model 2), and local sample (Model 3) to compare the joint effects of social connections and sociodemographic variables on the health of migrant and native adolescents. The models were designed in three steps. The first step of Model 1 consisted of age, gender, location of residence, and migrant status, while that of Models 2 and 3 included age, gender, and location of residence. The second step was included parental education, occupations, and family income. The third step added social connection variables. The latter two steps were the same in all three models. Significance and goodness of fit were tested for each final model. All analyses were performed using SPSS 16.0 software (IBM SPSS, Armonk, New York).

## Results

### Participation and socio-demographic characteristics

A total of 1,247 junior high school students received the survey, and the response rate was 98.5%; 213 invalid questionnaires were discarded. The analysis included 1,015 surveys (55.1% boys, 44.9% girls). The average age of participants was 13.6 years (SD = 1.09, range = 11-17). Table 1 shows significant diversity in demographics and SES in the sample, which included 712 migrants (70.1%) and 303 local adolescents (29.9%). Grade and gender were similar for the two groups, but the average age of migrants was older than natives. 67.0% of the migrants were from rural China's inland provinces, with the rest from other urban areas. Compared with local students, migrants were more likely to be from rural areas, and their parents were less likely to be homeowners in this area of Shanghai (*P *< 0.001). Migrants had lower family income, and their parents had less education and low-status occupations than native youth (*P *< 0.001).

**Table 1:** Comparison of socio-demographic characteristics between migrant and local adolescents

Characters	Migrant sample (n = 712)	Local sample (n = 303)	*Chi/t*-test
Gender (Female; %)	44.5	45.9	0.157

Age (Year, Mean ± SD)	13.8 ± 1.1	13.2 ± 1.0	9. 014***

Grade (%)			5.627

*6*	41.4	48.8	

*7*	31.7	30.0	

*8*	26.8	21.1	

Residence location (Village/suburb; %)	67.0	29.0	124.0***

Living in one's own house (Yes; %)	14.2	94.7	583.8***

Family income (RMB/month; %)			80.02***

*< 3,000*	34.4	18.5	

*3,000-4,999*	46.2	36.0	

*5,000-10,000*	13.3	35.3	

*> 10,000*	6.0	10.2	

Parental education (Father/mother; %)			147.6/212.2***

*Middle school or less*	71.8/83.6	36.0/38.9	

*High school or professional school*	21.2/11.2	31.7/31.4	

*College or above*	7.0/5.2	32.3/29.7	

Parental occupation (Father/mother; %)			94.51/82.49***

*Low*	48.9/54.1	16.5/26.1	

*Middle*	43.1/42.7	68.0/61.4	

*High*	8.0/3.2	15.5/12.5	

*P < .05, **P < .01, ***P < .001

### Comparison of health outcomes and social connections between migrant students and local peers

Table 2 shows sample statistics of dependent and key independent variables, stratified by migrant status. In general, migrant adolescents reported a higher proportion of self-rated excellent or good physical health (*P *< 0.05), and significantly less poor health compared to their native peers (*P *< 0.01). Migrants had a slightly higher level of depressive symptoms (*P *< 0.05), and significantly lower self-esteem than local adolescents (*P *< 0.001). They also had poorer social connections compared with nonimmigrant peers. There were significant disparities between migrants and natives in social cohesion (*P *< 0.001), family cohesion (*P *< 0.001), and peer association (*P *< 0.01). The only exception was in teacher-student relations.

**Table 2:** Comparison of predictors and dependent variables among migrants and local counterparts

Variables	Migrant sample (n = 712)	Local sample (n = 303)	*Chi*/*t- *test
Social connections (Mean ± SD)			

Family cohesion	66.0 ± 11.4	70.3 ± 13.3	-4.936***

Peer association	25.9 ± 4.8	26.9 ± 4.4	-3.451**

Teacher relations	19.8 ± 3.9	19.7 ± 4.0	0.257

Social cohesion	21.8 ± 4.0	23.0 ± 4.2	-4.347***

Self-rated physical health (%)			

Excellent/good	60.5	51.8	6.629*

Poor	8.6	14.2	7.31**

Mental wellbeing (Mean ± SD)			

Depression	17.2 ± 9.5	15.6 ± 9.3	2.39*

Self-esteem	28.3 ± 4.4	29.9 ± 5.0	-5.061***

*P < .05, **P < .01, ***P < .001

### Effects of social connections, demographics factors, and socioeconomic variables on three health outcomes

Tables 3, 4 and 5 show results from hierarchical regression models of the three health outcomes. Across the three models (total, migrant, and local) of Tables 3, gender was significantly associated with self-rated good physical health; girls reported substantially lower odds of having excellent/good physical health among all samples. The addition of SES variables showed that the father's education level was significant in the migrant sample (OR = 0.683, *P *< 0.05). Migrants who had fathers with middle level education (high school to some college) were more likely to report good physical health than those who had fathers with a high level of education (college or above) (OR = 3.482, *P *< 0.05). The effects of social connections on self-rated physical health are shown in Step 3 of Table 3. Migrant students with better peer relations (OR = 1.068, *P *< 0.05) and strong social cohesion (OR = 1.053, *P *< 0.01) were significantly more likely to report excellent/good physical health. Among local students, positive teacher relations (OR = 1.010, *P *< 0.05) and strong social cohesion (OR = 1.102, *P *< 0.05) were significantly associated with self-rated good physical. Step 3 of Model 1 shows migrant status as significant (OR = 1.051, *P *< 0.05). This outcome is consistent with crude statistics indicating that migrants are significantly more likely to report self-rated excellent/good physical health than their local counterparts.

**Table 3:** Logistic regression coefficients of demographic factors, socioeconomic status, and social connections on adolescents' self-rated good/excellent physical health

Predictors	Model 1 (n = 1015)	Model 2 (n = 712)	Model 3 (n = 303)
	Adjusted OR (95%CI)	Adjusted OR (95%CI)	Adjusted OR (95%CI)

Step 1			

Demographics factors			

Age (mean)	1.029(0.927-1.212)	1.199(0.973-1.462)	1.015(0.419-1.127)

Gender (Female)	0.587(0.423-0.815)**	0.642(0.469-0.878)**	0.534(0.330-0.864)*

Residence location (Town/city;%)	1.037(0.780-1.565)	0.844(0.608-1.173)	1.018(0.521-1.283)

Migrants or not (yes)^1^	1.032(0.413-1.055)		

Step 2			

Socioeconomic status			

Father's education (%)^2^	0.544(0.384-0.770)*	0.683(0.265-0.897)*	1.136(0.778-1.659)

Mother's education (%)	0.886(0.618-1.272)	0.782(0.527-1.161)	0.974(0.679-1.397)

Parental occupations (%)	1.059(0.807-1.389)	1.177(0.911-1.521)	1.047(0.890-1.224)

Family income (%)	1.036(0.838-1.282)	1.019(0.834-1.245)	1.011(0.730-1.400)

Step 3			

Social connections			

Family cohesion(mean)	1.005(0.982-1.018)	1.010(0.994-1.027)	0.994(0.971-1.017)

Peer associations(mean)	1.056(1.012-1.107)*	1.068(1.018-1.113)*	0.973(0.905-1.047)

Teacher relations(mean)	1.013(0.964-1.068)	1.024(0.974-1.077)	1.010(1.003-1.053)*

Social cohesion(mean)	1.064(1.015-1.125)**	1.053(1.025-2.035)**	1.102(1.014-1.198)*

OR = odds ratios; CI = confidence intervals; *P < .05, **P < .01, ***P < .001

^1^In step 3 of Model 1, the regression coefficients is significant as 1.051(1.024-1.217)*

^2 ^In step 2 of Model 2, college or above education level as reference group in further analysis, the regression coefficients is significant as 3.482(1.728-6.885)*

**Table 4:** Hierarchical regression of self-esteem on demographic factors, socioeconomic status, and social connections among migrant and local adolescent

Predictors	Model 1 (n = 1015)		Model 2 (n = 712)		Model 3 (n = 303)	
	*B*	*R2*	*B*	*R2*	*B*	*R2*

Step 1						

Demographics factors		0.047		0.017		0.038

Age (mean)	0.019		0.001		0.070	

Gender (Female)	0.093**		0.060		0.171**	

Residence location (Town/city; %)	0.113**		0.120**		0.072	

Migrants or not (yes)^1^	-0.130***					

Step 2						

Socioeconomic status		0.103		0.115		0.089

Father's education (%)	0.158***		0.156**		0.243**	

Mother's education (%)	0.088*		0.188***		0.041	

Parental occupations (%)	0.139***		0.138***		0.084	

Family income (%)	0.167***		0.251***		0.053	

Step 3						

Social connections		0.376		0.370		0.430

Family cohesion(mean)	0.231***		0.235***		0.240***	

Peer associations(mean)	0.145***		0.170***		0.114	

Teacher relations(mean)	0.175***		0.087*		0.335***	

Social cohesion(mean)	0.156***		0.190***		0.089	

*P < .05, **P < .01, ***P < .001

^1^In step 3 of Model 1, the regression coefficients is significant as -0.093*

**Table 5:** Hierarchical regression of depression on demographic factors, socioeconomic status, and social connections among migrant and local adolescent

Predictors	Model 1 (n = 1015)		Model 2 (n = 712)		Model 3 (n = 303)	
	*B*	*R2*	*B*	*R2*	*B*	*R2*

Step 1						

Demographics factors		0.025		0.019		0.025

Age (mean)	0.142***		0.134***		0.141*	

Gender (Female)	0.022		0.30		0.001	

Residence location (Town/city; %)	0.009		-0.014		0.065	

Migrants or not (yes)	-0.042					

Step 2						

Socioeconomic status		0.047		0.045		0.061

Father's education (%)	-0.107*		-0.114*		-0.146	

Mother's education (%)	-0.127**		-0.140**		0.085	

Parental occupations (%)	-0.104**		-0.070		-0.168**	

Family income	-0.067		-0.103*		0.028	

Step 3						

Social connections		0.435		0.418		0.509

Family cohesion(mean)	-0.238***		-0.251***		-0.201***	

Peer associations(mean)	-0.330***		-0.358***		-0.280***	

Teacher relations(mean)	-0.144***		-0.073*		-0.306***	

Social cohesion(mean)	-0.117***		-0.122**		-0.108	

*P < .05, **P < .01, ***P < .001

Table 4 shows the results of regression analyses on self-esteem. In Model 2, location of residence, SES, and social cohesion were all significantly correlated with self-esteem scores. Migrant adolescents with strong social cohesion and higher SES reported better self-esteem. In migrants only, peer association (*β *= 0.170, *P *< 0.001) and social cohesion (*β *= 0.190, *P *< 0.001) were significantly correlated with self-esteem. In Model 3, there was a positive association between female gender and self-esteem (*β *= 0.171, *P *< 0.01). Family cohesion was significantly and positively associated with self-esteem scores in Models 2 and 3 (*β *= 0.235; *β *= 0.240, *P *< 0.001). Teacher relations were a more significant factor in Model 3 (*β *= 0.335, *P *< 0.001) than in Model 2 (*β *= 0.087, *P *< 0.05). In addition, migrant status was significantly associated with self-esteem in Model 1 in steps 1 (*β *= -0.130, *P *< 0.001) and 3 (*β *= -0.093, *P *< 0.05). These outcomes support crude statistics showing substantially lower self-esteem among migrant adolescents than local peers.

Table 5 shows results from a parallel regression analysis, with depression as the dependent variable. According to step 1, there was a significant association between age and depression among the three Models. This indicates that older adolescents reported more depressive symptoms. In step 2, there was a negative association between SES variables and depression. For instance, parental education level and family income were significant in Model 2, while parental occupation status was significant in Model 3. Step 3 of Table 5 showed negative associations between family cohesion (*P *< 0.001), peer associations (*P *< 0.001), teacher relations (*P *< 0.05), and depression in Models 2 and 3. In Model 2, social cohesion (*β *= -0.122, *P *< 0.01) was significant for migrants only. Thus, there was a negative association between social cohesion and depressive symptoms in migrant adolescents. Further analyses of regression coefficients showed that family cohesion (*β *= -0.251 vs. *β *= -0.201) and peer association (*β *= -0.358 vs. *β *= -0.280) were more important in Model 2 than in Model 3, while teacher relations (*β *= -0.306, *P *< 0.001 vs. *β *= -0.073, *P *< 0.05) were more significant in Model 3. In Model 1, there was no significant association between migrant status and depression. This suggests that the difference in depression between migrants and local peers became insignificant after all proposed mediators were taken into account.

## Discussion

Our study showed that original residents gained a significant advantage from SES, which is consistent with the prior research [5-7]. The three indices of migrant SES were worse than that of natives (*P *< 0.001). This may be largely due to the substantial proportion of rural-to-urban migrants in our study, whose SES and personal resource were generally poor. Perhaps as a consequence, migrant adolescents showed worse social connections than their local peers, with significant disparities in social cohesion, family cohesion, and peer associations. These outcomes are consistent with those from prior studies in China [6,24,25]. Although we did not find a significant difference in student-teacher relations between migrant and native adolescents, local boys reported the lowest scores in teacher relations. A plausible explanation is that migrants and girls are generally considered more likely to obey rules, and respect teachers compared with local students and boys [36].

Our data support prior research showing that migrants have significantly lower self-esteem than local adolescents [9]. The present study also shows that all indicators of SES and social connection indices were positively correlated with self-esteem scores in migrants. The effects of SES among local students were not as significant as migrants. Namely, poor social connections and low family SES may affect the self-confidence and self-esteem of migrant adolescents particularly. Differences in the values and patterns of behavior between the children of migrant workers in China and their urban peers (e.g., heavy regional accent, out-of-date clothing, and communication difficulties) might lead to social discrimination that contributes to a lack of self-confidence and social isolation among migrant children and adolescents [25,39].

Age was significantly associated with depression among migrants. This outcome is consistent with previous research that identified age as a risk factor for psychological well-being in migrant children and adolescents [6]. Although migrant adolescents showed more depressive symptoms than local youth, the difference diminished when sociodemographic factors were considered. However, the difference in self-esteem between the two samples still persisted. Thus, our data partially support prior research showing that the internal migrant adolescents have poorer psychological health than their local peers in China.

Interestingly, migrant adolescents' self-rated physical health was generally better than that of local peers. This is consistent with the healthy migrant effect, which has been described in many Western countries [40]. Evidence from Chinese adult migrants also demonstrates the healthy migrant phenomenon [17,41]. One explanation is the health selection hypothesis, which emphasizes that healthier individuals are more likely to migrate [40]. Another hypothesis is that the migrants are more likely to return to their places of origin when they have a serious illness [42]. It is noted that girls are more likely than boys to report fair/poor physical health. This result might reflect differences between migrants and native residents, and boys and girls in their appraisals of health and attitudes about ill health.

Self-rated poor health by migrant adolescents was related to the father's education, i.e., middle or high level. In this study, a middle education level had a protective effect. Miao [43] suggested that high parental education level might be a protective factor for parents themselves, but a risk factor for the health of migrant adolescents. Most likely, parents with a high education level expect academic success from their children; doing poorly in school could cause families to lose face. In China, academic success is a high priority for parents and children. Parents, in turn, pursue all options to ensure that their children work hard and strive for academic excellence [44]. In rural areas, fathers generally have more education than their wives, and are more likely to supervise their children's academic achievement. The outcome might be more academic pressure accompanied by a high level of distress in their offspring. The parental education effect may differ between migrant and local families. In the modern Chinese family, well-educated mothers who become stricter and stricter with their children--"Chinese tiger moms"-- are very popular in urban areas [45]. Our data suggest that the self-rated physical health of adolescents is a complex and multifaceted phenomenon. The results of our study merit further exploration.

Collectively, this report showed that layered social connections are linked to higher self-esteem and lower levels of depression among migrants. This suggests the importance of social connections to mental health in adolescents. We found a stronger sense of peer association and social cohesion to be particularly beneficial for migrants in all three health outcomes. This suggests that the two factors may be particularly protective for immigrants. Migration is characterized by a loss of friendships and challenges to the establishment of new ties. Those who form and maintain good peer relationships and feel more connected with others are likely to have better psychological well-being [25]. Strong social cohesion may mitigate the effects of a lack of other socioeconomic resources for certain populations [17]. Family cohesion was associated with both depressive symptoms and self-esteem among all adolescents. This finding confirms the well-studied importance of family relations for adolescent mental health in immigrant and non-immigrant groups [46-48].

Our results should be interpreted with caution due to methodological limitations of the study. Its cross-sectional design preempts the ability to establish cause-effect relationships. The sample of migrants all came from the suburban public schools in Shanghai, which have a mainly middle-low SES population, specifically among rural-to-urban migrants. That both migrant and local residents were recruited from a single major metropolitan area limits the generalizability of the results. Self-reported demographic and health data might be biased. This is especially true on measures of self-rated physical health [49,50], which might be subject to reverse causation.

## Conclusions

Limitations notwithstanding, this study contributes to the literature by examining the relationships between layered social connections and health issues among Chinese migrant and local adolescents that have been neglected in prior studies. Data show that migrants have a significant disadvantage in psychological well-being and social connections; that family cohesion is an important factor for the psychological well-being of all adolescents; and that social cohesion and peer associations play a strong role, especially as they relate to migrant health. These findings indicate the importance of strengthening the interpersonal systems of adolescents. In particular, social cohesion and positive connections with parents and peers are significant protective factors that can enhance the mental health of migrant adolescents in China. For instance, parents can pay more attention to their children' psychological well-being, and enhance family cohesion. Educators may encourage young migrants to actively engage local peers and organize support groups for those who feel incompetent or inferior to local peers. Such groups should supply information and teach skills that improve interactions with urban residents, and help migrant youth make a more balanced appraisal of their own abilities and qualities [25]. Local organizations can help by organizing social workers and volunteers from nearby universities and colleges, thereby creating a bigger and broader safety net. It may also be important for the government and relevant organizations to study the issue and develop strategies for addressing the needs of migrant adolescents at a local level.

## Competing interests

The authors declare that they have no competing interests.

## Authors' contributions

ZHM draft the manuscript and conducted the study. XDZ conceived and planned the study and participated in introduction, results interpretation and discussion of this manuscript. All authors have read and approved the final manuscript.

## Pre-publication history

The pre-publication history for this paper can be accessed here:

http://www.biomedcentral.com/1471-2458/12/97/prepub
